# Open-joint1Read before the March meeting of the Chicago Veterinary Society.

**Published:** 1897-05

**Authors:** A. H. Baker

**Affiliations:** Chicago, Ill.


### OPEN-JOINT.[1]

By A. H. Baker, D.V.S.,
CHICAGO, ILL.

Open-joint is one of the most serious and one of the most common traumatic ills we meet with in everyday practice, and one that is skimmed over lightly or not noticed at all by authors on veterinary surgery, and, I feel free to say, one in which is employed the most old-fashioned and generally useless treatment of any. We notice veterinarians using the same remedy and general procedure to-day that our forefathers used, in many cases not even giving the same usually good treatment that ordinary simple traumatisms receive. Poultices and blisters seem to be the popular remedies of many of our best men and teachers in veterinary schools.

With a view of recalling to mind the serious nature of the trouble, I will briefly run over the pathogenesis of it. An open-joint is a wound where the skin, the binding and capsular ligaments, and the synovial membrane are ruptured, allowing escape of the synovia and ingress for germs of various kinds that cause inflammation of the tissues, especially synovitis and oftentimes arthritis. The case runs through four well-defined stages: First, a flow of pure synovia in limited quantity. Second, as the inflammation develops the flow of synovia increases, and, if confined so as to accumulate, it coagulates in the form of an amber-colored, odorless clot; the joint swells, becomes painful; fever develops, the pulse is hard and increased in frequency, the animal is restless and inclined to keep the affected limb in nearly constant motion if the trouble is below the elbow or stifle; he usually persistently stands, becomes tucked up, and emaciation is rapid; after a couple of days more or less pus is mixed with the discharge. Third, the articular cartilage becomes involved, suppurates, ulcerates, and becomes absorbed; the discharge in this stage is offensive and occasionally streaked with blood; the limb swells extensively, and numerous abscesses form around the affected joint, rupture and form sinuses; the swelling becomes indurated; he never bears weight upon the limb, usually ruins the other leg, and dies in the course of two to six weeks. If it runs a somewhat milder course, with less swelling, suppuration, and induration, it runs into the fourth stage, in

[1] Read before the March meeting of the Chicago Veterinary Society.

which the articular ends of the bones become involved in a rarefying ostitis, with a liberal exudation of lymph, which coagulates, organizes, and anchylosis is the result, more or less ruining his usefulness, according to which joint is affected and the class of work he is adapted to. The leg is always permanently enlarged, with considerable ossification of the inflammatory deposits.

In suggesting a rational plan for treatment I bear in mind that the immediate cause of all this trouble can be summed up in one word—*sepsis*, and that the secret of success in treating an open-joint lies also in one word—*antisepsis.*

Septic germs find a fertile field in such a wound, and the synovial membrane, being apparently very sensitive, yields quickly to the irritation produced by them. The germs may enter at the time of the accident, or afterward, and it is these that we have to fight from the start. Poultices are an abomination, no matter how well they may be cooked with a view to sterilize them, or how well the attempt may be made to sterilize them by the use of antiseptics, for germs will work their way in between the poultice and the skin, and often find a fertile soil in the poultice itself. Blisters are nearly as bad. They serve to increase the pain by producing extension of the inflammation to the more superficial parts without reducing it in the joint. They tend to close the external opening by the swelling they produce; this, however, does not lessen the activity of the destructive process going on inside, but rather tends, in my opinion, to promote it and to increase the liability to form abscesses and sinuses in the surrounding tissues. Treatment, to be rational, must be directed toward reducing the inflammation by removing the cause, viz., destroying the germs that have entered the wound, and allowing granulation to go on in a natural way.

The first thing to do is to remove the hair as closely as possible from a considerable surface, remove shreds of lacerated tissue, if any, wash the wound with soap and water, then irrigate it thoroughly by syringing with an antiseptic lotion. Do not introduce the nozzle of the syringe into the wound, but, holding it a little distant from it, shoot the lotion into it with a little force for ten or fifteen minutes; then do it up by covering it freely with an antiseptic powder—for instance, iodoform, powdered boric acid, and starch in equal parts—and apply a good-sized pad of absorbent cotton over it, and hold it in place with a bandage. Dress it night and morning. This is imperative on account of the necessity of removing the synovial clot that forms on the cotton. If the case does not do well with this treatment, it is because the wound is not aseptic, and in such a case there is the continuous motion spoken of induced by the pain. In such a case more heroic surgery is needed. The wound, probably, is too small; enlarge it with a probe-pointed bistoury half an inch each way upward and downward, making a V-shaped opening; irrigate it again, no matter if the lotion enters the joint. Use for this purpose bichloride of mercury, 1 to 2000, for ten minutes, then reduce it to 1 to 5000 to rinse out the stronger solution; then do it up as before, and put on an iron brace to stiffen the leg, to prevent motion in the affected joint. The necessity of this should be anticipated, and the proper shoe applied to the foot before too much soreness has developed. Immobility in an open-joint is a *sine qua non* to success. The brace is the only effectual means of accomplishing this. The old-fashioned splint is a failure. The brace does not interfere with the application of the dressings, nor does it chafe the leg, if properly padded.

The brace is made of half-inch square iron, with a hook on the bottom to hook into a hole in the bar of a foreshoe, being bent to fit the outline of the back of the leg, running up to the middle of the arm, with a piece of broad band-iron fastened to it at the top to clasp the arm. One or two leather straps are fastened to the brace between the band and the foot, to keep the leg in its natural position. The brace for a hindleg is hooked into the toe of the shoe, and is bent to follow the outline of the front of the leg up to about four inches above the hock, with similar band and straps as for the forelimb. All of the bearings between the brace and the leg should be well padded.

In cases of open-shoulder, hip-joint, or stifle-joint the swelling is apt to be excessive. The wounds usually require enlarging with a knife to afford ample opportunity for thorough irrigation. The wound is usually deep on account of the swelling. This condition enables us to insert an antiseptic plug—not to prevent the flow of the discharge, but to keep out germs—composed of solid extract of belladonna, tannic acid, and carbolic acid, one drachm each; wheat-flour, two ounces; water, q.s. to form a paste. Press a little of this well down into the wound, then foment the swelling with warm water persistently, and repeat three times a day.

Open navicular joint is a very common case in city practice, and one that very often results disastrously, either in death of the animal or permanent lameness. When a nail-prick is found to be an open-joint, the soaking and poultices that had been used up to that time should be discontinued, the hoof and frog freely pared away, and the wound freely exposed and enlarged to permit of irrigation. After doing this do it up in a 3 per cent. solution of carbolic acid, with oakum for a pack. Let it cover the sole, heels, and coronet. Keep this wet continually by pouring the lotion freely into the pack several times daily. Renew the pack night and morning to remove the oakum soiled with the synovia and pus. Instead of hot fomentations, whether there is swelling or not, ice-packs or ice-water may be applied and kept up persistently until healing has advanced to a point where there is no more danger of severe inflammation.

Open-joints treated in this way almost invariably make good recoveries, the inflammation subsides, the flow of synovia gradually stops, the wound granulates and heals by second intention without suppuration.

If some swelling and considerable lameness persist, especially in the foot, repeated cantharidean blistering and long rest will usually lead to resolution.

If the horse will lie down, I prefer to have him do so; but if he persistently stands, put him into slings during the more acute stage.

Open-joints that occur as a result of necrosis and sloughing beneath the skin in cases of contusions usually prove fatal on account of the extensive disorganization.

